# The utility of anti-SOX2 antibodies for cancer prediction in patients with paraneoplastic neurological disorders

**DOI:** 10.1016/j.jneuroim.2018.11.003

**Published:** 2019-01-15

**Authors:** Paul Maddison, Maarten J. Titulaer, Jan J. Verschuuren, Paul Gozzard, Bethan Lang, Sarosh R. Irani, Lidia Sabater, Francesc Graus, Andrea Murray, Caroline J. Chapman

**Affiliations:** a Department of Neurology, Nottingham University Hospitals NHS Trust, Queen's Medical Centre, Nottingham NG7 2UH, UK; b Department of Neurology, Erasmus University Medical Centre, Rotterdam, The Netherlands; c Department of Neurology, Leiden University Medical Centre, Leiden, The Netherlands; d Department of Neurology, Sheffield Teaching Hospitals NHS Foundation Trust, Royal Hallamshire Hospital, Sheffield S10 2JF, UK; e Nuffield Department of Clinical Neurosciences, University of Oxford, West Wing, John Radcliffe Hospital, Oxford OX3 9DS, UK; f Neuroimmunology Program, Institut d'Investigació Biomèdica August Pi i Sunyer (IDIBAPS), University of Barcelona, Barcelona, Spain; g Oncimmune, Ltd., Nottingham, UK; h Department of Pathology, Nottingham University Hospitals NHS Trust, Queen's Medical Centre, Nottingham NG7 2UH, UK; i Service of Neurology, Hospital Clinic, Barcelona, Spain

**Keywords:** SOX2, Antibodies, Paraneoplastic, Lambert-Eaton myasthenic syndrome, Small-cell lung cancer, ELISA, enzyme linked immunosorbent assay, LE, limbic encephalitis, LEMS, Lambert-Eaton myasthenic syndrome, NMDA-R, *N*-methyl-d-aspartate receptor, OD, optical density, OMS, opsoclonus-myoclonus syndrome, PCD, paraneoplastic cerebellar degeneration, SCLC, small-cell lung cancer, SOX2, Sry-like high-mobility group box protein 2, SSN, subacute sensory neuronopathy, VGCC, voltage-gated calcium channel

## Abstract

Antibodies to SOXB1 proteins in patients with paraneoplastic disorders are associated with small-cell lung cancer (SCLC), particularly in Lambert-Eaton myasthenic syndrome (LEMS). We aimed to establish if SOX2 antibodies could be used to identify SCLC and other tumours found in a range of paraneoplastic disorders and controls.

SOX2 antibodies were detectable in 61% of patients with LEMS-SCLC, and in other paraneoplastic disorders, such as opsoclonus-myoclonus and paraneoplastic cerebellar degeneration, only when there was an underlying SCLC.

SOX2 antibodies are specific (>90%) markers for SCLC, but are rarely found in patients with other tumours, whether neurological symptoms are present or not.

## Highlights

• SOX2 antibodies only seen in small-cell lung cancer (SCLC), not other tumours
• 61% of patients with Lambert-Eaton syndrome and SCLC have SOX2 antibodies.
• Patients with paraneoplastic disorders only have SOX2 antibodies if SCLC is present.

## 1 Introduction

Paraneoplastic neurological disorders (PNDs) are a diverse group of conditions in which distant tumour effects are thought to underlie the neurological presentation. These effects are generally thought to be due to an immune-mediated process with cell-mediated and humoral components. In a Europe-wide review, small-cell lung cancer (SCLC) was the commonest associated tumour in patients with PNDs, (present in over a third of cases), with non-small-cell lung cancer, thymoma, lymphoma, ovarian and breast cancer found in a further 37% (Giometto et al., 2010).

Many PNDs are associated with specific onconeural antibodies against intracellular antigens (Hu, Yo, Ri, CV2 (CRMP5), amphiphysin, Ma2), but there is considerable diversity such that no single antibody is associated with only one type of neurological presentation or underlying tumour (Pittock et al., 2004). For example, anti-Hu antibodies are found in patients presenting with a range of PNDs such as subacute sensory neuronopathy (SSN), limbic encephalitis (LE), paraneoplastic cerebellar degeneration (PCD), but also in patients with LEMS where the presence of Hu antibodies may be a marker of an underlying small-cell lung tumour (Titulaer et al., 2009). In addition, although patients with particular paraneoplastic neurological phenotypes often develop specific tumours (e.g. SCLC found in over 90% of paraneoplastic LEMS (Titulaer et al., 2011a) and ovarian teratoma in over 90% of patients with anti-NMDA-R encephalitis (Titulaer et al., 2013)), this is not always the case and some PND presentations can be associated with a number of different cancers (e.g. LE seen with tumours of the lung, testis, and breast, and also with Hodgkin's lymphoma, teratoma, or thymoma) (Gozzard and Maddison, 2010).

Antibodies to certain antigens have been found to be useful markers of SCLC, most notably SOXB1 group antigens (SOX1, SOX2, SOX3), Sry-like high mobility group box proteins that are expressed in neuronal precursor cells and adult human cerebellum (Alcock et al., 2009). In the context of PNDs, a study of 86 patients with LEMS found antibodies to SOX proteins in 67% of 43 LEMS patients with an underlying SCLC, but only in 4.6% (2/43) of non-tumour LEMS patients (Titulaer et al., 2009). In patients with PCD and SCLC, SOX1 antibodies were detected in 80% of patients with coexistent LEMS, but only 38% of patients with pure PCD (Sabater et al., 2013). SOX1 antibodies were found less frequently in patients with neuropathies (5/32, 16%), most often with associated SCLC and anti-Hu antibodies, although a similar number (4/22, 18%) had no underlying tumour (Tschernatsch et al., 2010). Recently, in a larger study of patients with additional neurological presentations, SOX1 antibodies were found in 2/247 (0.8%) patients with multiple sclerosis, 2/185 (1.1%) with neuropathy of unknown cause and 11/837 (1.3%) with suspected PNDs, most of whom had no underlying tumour, and none with SCLC (Berger et al., 2016).

Prospective studies in SCLC patients have indicated that the presence of SOX2 antibody on its own is not related to a distinct pure neurological phenotype, suggesting that SOX antibodies may be of most use in predicting the presence of an underlying SCLC with high specificity (Maddison et al., 2010; Chapman et al., 2011). As a number of patients may also present with neurological clinical features identical to classical PNDs such as LE or cerebellar ataxia, without an underlying tumour, we therefore aimed to establish firstly if SOX antibodies could be used to distinguish between paraneoplastic and non-paraneoplastic presentations in patients with a range of PNDs and neurological mimics, and secondly whether raised levels of SOX antibodies were present in a number of other common tumour types seen in PNDs, such as thymoma, teratoma, breast or ovarian cancer.

## 2 Methods

### 2.1 Patients

Sera were obtained from a number of patient groups (Table 1).

**Table 1:** Patient group clinical details.

Clinical groups	Number	Median age years (range)	Percent female	SOX2 positivity
Neurological patients	313			
LEMS-SCLC	61	60.5 (35–86)	34%	61%
LEMS no tumour	65	53 (14–80)	48%	6%
OMS-lung cancer^a^	9	58 (46–72)	11%	33%
OMS no tumour	10	32 (21–46)	70%	10%
PCD^b^	20	60 (48–79)	50%	50%
Idiopathic ataxia^c^	20	59 (35–84)	60%	0%
NMDA-R antibody encephalitis with teratoma	8	25 (20–40)	88%	0%
NMDA-R antibody encephalitis with no tumour	10	25 (12–63)	80%	0%
Paraneoplastic limbic encephalitis^d^	20	63 (3–78)	35%	30%
Subacute sensory neuronopathy^e^	45	68 (40–89)	64%	42%
Myasthenia with thymoma	28	57 (25–80)	50%	0%
Myasthenia with no tumour	17	31 (16–49)	89%	0%

Patients without neurological symptoms	1090			
SCLC	259	66 (33–87)	49%	30%
Non-SCLC	238	66 (43–85)	22%	6%
Breast cancer	75	60 (20–88)	100%	0%
Ovarian cancer	104	58 (27–81)	100%	4%
Healthy controls	414	63 (17–88)	77%	2%

LEMS, Lambert-Eaton myasthenic syndrome; SCLC, small-cell lung cancer; OMS, opsoclonus-myoclonus; PCD, paraneoplastic cerebellar degeneration; NMDA-R, *N*-methyl-d-aspartate receptor.

a Six patients had SCLC, three non-SCLC.

b 16 patients also had LEMS-SCLC, one had LEMS and no tumour, one had Hu antibodies and SCLC, and two had Yo antibodies and breast or ovarian cancer.

c Seven patients had gluten ataxia.

d 18 patients had Hu antibodies (10 with SCLC, 4 CT/PET evidence of lung cancer, one neuroblastoma, one no cancer detected), one GABA_B_ antibodies (with SCLC) and one had no detectable onconeural antibodies (with SCLC).

e All 45 patients had Hu antibodies (32 with SCLC, 5 no cancer detected, 3 CT/PET evidence of lung cancer, 2 non-SCLC, 1 each of oesophagogastric adenocarcinoma, metastatic endocrine carcinoma and prostatic adenocarcinoma).

As part of an ongoing prospective study of patients with paraneoplastic disorders, 289 unselected patients with biopsy-proven SCLC, were recruited at the time of tumour diagnosis from hospitals in the Trent region of the UK between 2005 and 2011 (Gozzard et al., 2015). All prospectively recruited SCLC patients underwent full neurological evaluation and examination, and follow-up assessments were undertaken for those who subsequently developed neurological symptoms. Of the initial 289 patients recruited, 259 had no associated paraneoplastic neurological symptoms. Sera from 238 patients with non-SCLC, 75 patients with breast cancer (both ductal carcinoma in situ, *n* = 39, and stage 1 invasive disease, *n* = 36), and 104 patients with ovarian cancer, none of whom had neurological illness, were also provided through studies by University of Nottingham (Boyle et al., 2011). Control sera were obtained from healthy individuals from the Trent region of the UK and were matched for age, gender and smoking status with the cancer patients: all control subjects were reported as having no previous or current history of cancer, and no underlying neurological conditions.

Additional sera were obtained from 20 patients with PCD, of whom 17 had additional symptoms of LEMS (16/17 with SCLC), two had anti-Yo antibodies with ovarian or breast cancer, and one had pure PCD and SCLC; and from 20 patients with idiopathic late-onset cerebellar ataxia (Hadjivassiliou et al., 2008). Sera were obtained from 19 patients with opsoclonus-myoclonus (9 paraneoplastic: 6 SCLC, 3 non-SCLC; and 10 non-paraneoplastic, referred to one of the authors, (FG, Barcelona, Spain). Initial diagnostic sera from 18 patients with NMDA-R-antibody associated encephalitis (7 ovarian teratoma, 1 testicular teratoma; 10 non-paraneoplastic) and 45 patients with acetylcholine receptor antibody positive myasthenia gravis, (28 of whom had an associated thymoma), were also analysed.

Eighteen patients with anti-Hu antibody associated LE (10 SCLC, 4 CT/PET evidence of lung cancer, 3 no cancer detected and 1 neuroblastoma) and a further two paraneoplastic LE patients (both with SCLC, one with GABAb antibodies) were included. Sera were also obtained from 45 patients with anti-Hu antibody associated SSN (32 SCLC, 5 no cancer detected, 3 CT/PET evidence of lung cancer, 2 non-SCLC, 1 each of oesophagogastric adenocarcinoma, metastatic endocrine carcinoma and prostatic adenocarcinoma).

We also collected sera from 126 patients with LEMS (61 SCLC-LEMS, 65 non-tumour LEMS) from initial samples taken within 3 months of diagnosis referred to Oxford and Nottingham, UK, and Leiden, NL: 68 of the Dutch LEMS cases have been reported previously (Titulaer et al., 2009). Written, informed consent was obtained from all patients (Nottingham Research Ethics Committee 04/Q2404/100; Medical Ethics Committee of the Leiden University Medical Center).

Internationally accepted diagnostic criteria were used for LEMS (Oh et al., 2005; O'Neill et al., 1988), LE (Gultekin et al., 2000; Graus et al., 2004), OMS (Bataller et al., 2001), SSN and PCD (Graus et al., 2004), and myasthenia gravis (AAEM Quality Assurance Committee. American Association of Electrodiagnostic Medicine, 2001; Benatar, 2006). Neurological conditions were considered non-tumour-related if more than three years had elapsed from symptom onset without tumour detection in LEMS, and 4 years in the other neurological presentations, during follow-up screening (Titulaer et al., 2011b).

### 2.2 Antigen production and antibody detection

Recombinant SOX2 was produced and the presence of autoantibodies to SOX2 was evaluated using a semi-automated ELISA method as described previously (Chapman et al., 2011; Murray et al., 2010). Briefly, specimens were diluted (1 in 110) and then allowed to react with immobilised SOX2 at a range of different concentrations between 1.6 and 160 nM. Binding of autoantibody was reported using horseradish peroxidase labelled rabbit anti-human-IgG. Positive seroreactivity was defined as (a) having evidence of a dose response to the antigen dilution series and (b) an optical density (OD) value of the background corrected signal above a cut-off level set from the matched control data (mean + 3 SD).

Of the 126 serum samples from LEMS patients, 115 (91.2%) were also analysed in Leiden for SOX1 and SOX2 antibodies with ELISA and western blot, using techniques previously described (Titulaer et al., 2009). Additional western blots to SOX2 antigen were performed in Nottingham, UK, on any LEMS samples that gave discrepant results compared with the other laboratories. A final result was considered positive if either ELISA or western blot results were positive in more than one laboratory.

### 2.3 Statistical analysis

Comparisons between patient groups with and without associated tumours were performed using Fisher's exact test for contingency tables, Kruskal-Wallis test (for all six sets of SCLC-PND patient data) and Mann-Whitney *U* tests (for comparison of two unpaired groups).

## 3 Results

### 3.1 ELISA reproducibility

The ELISA using recombinant SOX2 developed in Nottingham was previously tested in reproducibility studies in over 1000 patients, confirmed in a validation set and found to have a very high reproducibility (Boyle et al., 2011; Murray et al., 2010). There were only 6/126 (4.8%) LEMS patients whose SOX2 ELISA results from Nottingham differed from those performed in duplicate in Leiden: subsequent SOX2 western blotting performed at Nottingham in three of these patients (where sufficient serum was available), who were initially negative on SOX2 ELISA, were all positive, improving concordance between laboratories in all cases. SOX1 and SOX2 ELISA results were markedly concordant (>98%): one LEMS-SCLC patient and one non-tumour LEMS patient had positive SOX2 antibodies but negative SOX1 antibodies. No LEMS patients were positive for SOX1 antibodies but negative for SOX2 antibodies.

### 3.2 Control patients

**Fig. 1:**
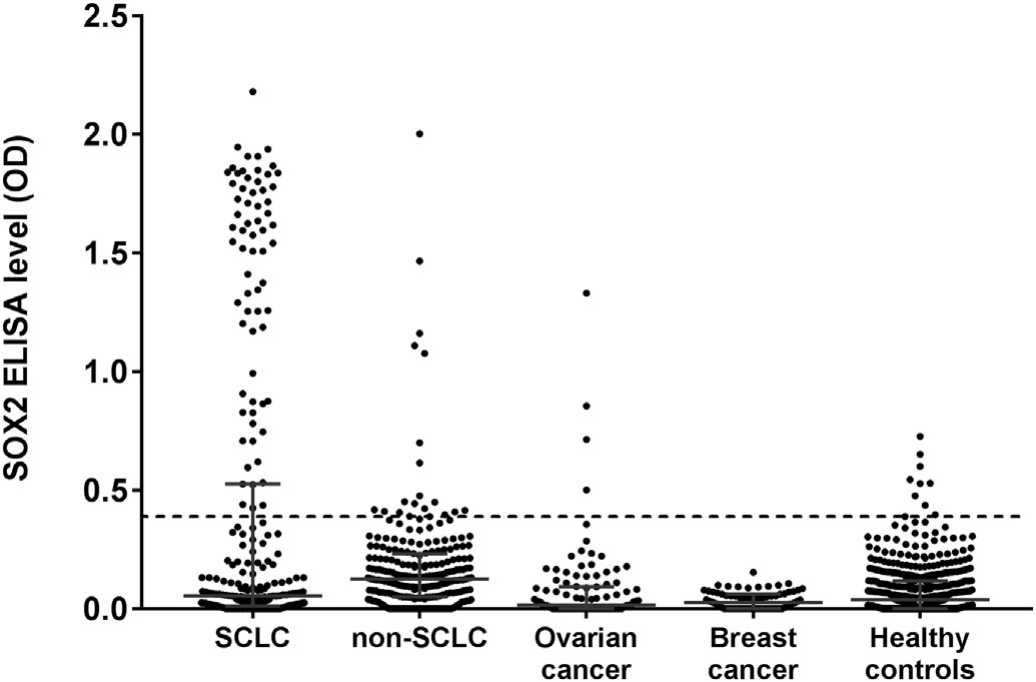
SOX2 antibody levels (in arbitrary units) in patients without neurological symptoms. OD = optical density; SCLC = small-cell lung cancer. Dotted line represents mean plus 3SD of healthy controls. Error bars represent medians ± interquartile ranges. Median levels higher in lung cancer (SCLC and non-SCLC) patients compared with all other groups (*P* < .001).

In patients without neurological symptoms, SOX2 antibodies were found in 77/259 (29.7%) patients with SCLC, but in only 15/238 (6%) of non-SCLC, 0/75 (0%) of breast cancer, 4/104 (4%) of ovarian cancer, and 8/414 (1.9%) of age matched healthy controls (Fig. 1, Table 1).

### 3.3 Patients with paraneoplastic disorders

Antibodies to SOX2 were detected by ELISA in 37/61 (61%) patients with LEMS-SCLC, and 4/65 (6%) patients with non-tumour LEMS (*P* < .0001) (Table 1). SOX2 antibodies were detected in 10/20 (50%) patients with PCD, exclusively with co-existent LEMS and SCLC, but not in one patient with pure PCD and SCLC, nor in idiopathic late onset cerebellar ataxia (0/20) (*P* = .0004). Two other patients with PCD and gynaecological cancer were both SOX2 antibody negative. We found SOX2 antibodies in 3/9 (33%) paraneoplastic OMS patients (all with SCLC), but in only 1/10 (10%) adult non-tumour OMS (*P* = .30). Five of 18 patients (28%) with anti-Hu antibody associated LE (4 SCLC, 1 no cancer detected) and one further LE patient with GABAb antibodies and SCLC were positive for SOX2 antibodies. In the anti-Hu antibody associated SSN patient group, 19/45 (42%) had SOX2 antibodies (16 SCLC, 2 CT/PET evidence of lung cancer, 1 metastatic oesophagogastric carcinoma). No patient with teratoma-related (0/8), or non-tumour associated (0/10) NMDA-R-antibody encephalitis, nor myasthenia with (0/28) or without (0/17) thymoma had SOX2 antibodies.

**Fig. 2:**
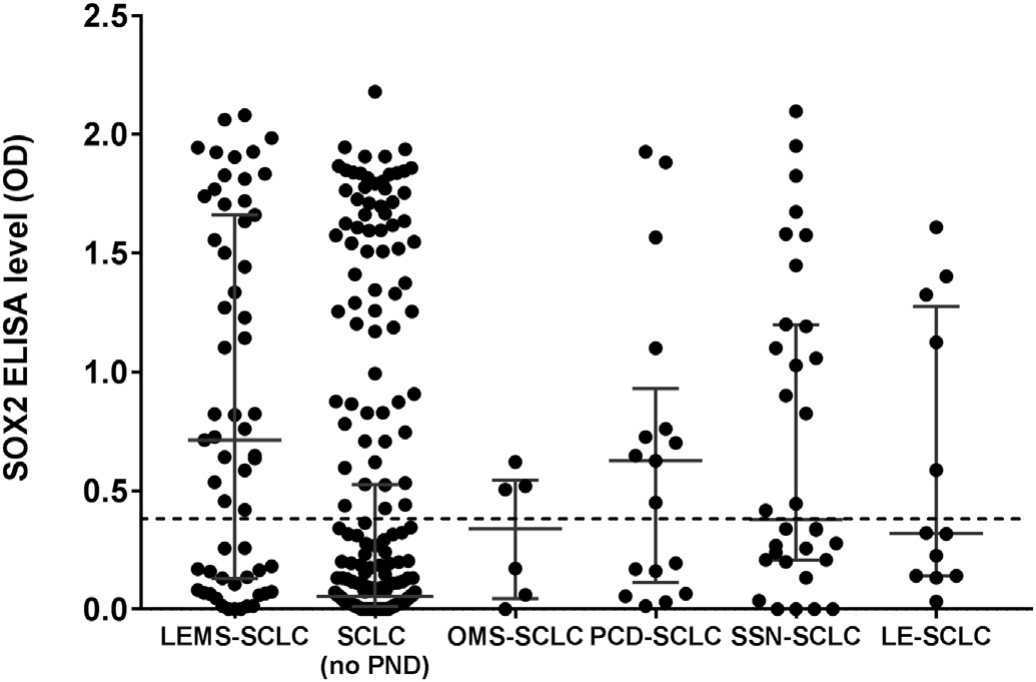
SOX2 antibody levels in patients with small-cell lung cancer and neurological paraneoplastic disorders. LE = limbic encephalitis; LEMS = Lambert-Eaton myasthenic syndrome; OD = optical density; OMS = opsoclonus-myoclonus syndrome; PCD = paraneoplastic cerebellar degeneration; SCLC = small-cell lung cancer; SSN = subacute sensory neuronopathy. Dotted line represents mean plus 3SD of healthy controls. Error bars represent medians ± interquartile ranges.

SOX2 antibody levels were significantly different across all six SCLC-PND patient groups (Fig. 2) (*P* < .0001, Kruskal-Wallis). Median SOX2 antibody levels (at the highest concentration of antigen analysed, 160 nM) were statistically higher in LEMS-SCLC (0.72 optical density, OD) than SCLC without PNS (0.10 OD) (P < .0001), OMS-SCLC (0.33 OD) (*P* = .05), SSN-SCLC (0.38 OD) (*P* = .044) or LE-PCD (0.32 OD) (*P* = .03),but more similar to PCD-SCLC (0.62 OD) (*P* = .48), the last of which almost all had associated LEMS (Fig. 2). The SOX2 levels in the four non-tumour LEMS patients with detectable SOX2 antibodies were all low-positive, ranging from 0.47 to 0.81 OD. The distribution of SOX2 antibody titres among the SCLC (no PND) patients was almost bimodal (Fig. 2), and statistically different in distribution from both the LEMS-SCLC (P < .0001) and PCD-SCLC (*P* = .007) patients (Kolmogorov-Smirnov two-sample test).

### 3.4 Clinical features of patients with SOX2 antibodies

Overall, there were no significant differences in terms of median age at symptom onset, percentage of female patients, or SCLC disease extent at cancer diagnosis, in all patients with SCLC and associated paraneoplastic neurological disorders (LEMS, OMS, LE, PCD, and SSN) in those who did or did not have SOX2 antibodies (Supplementary table 1, all *P* > .05). Patients with SCLC without neurological symptoms were similar in age, percentage female and SCLC disease extent at cancer diagnosis to those SCLC patients with neurological presentations whether they had SOX2 antibodies or not (Supplementary table 1).

## 4 Discussion

It was following the identification of a unique pattern of immunohistochemical staining of the Bergmann glia of the cerebellum in patients with paraneoplastic neurological disorders associated with SCLC (so-called anti-glial nuclear antibody, or AGNA) that the putative antigen, SOX1, was discovered to be the antigen recognised by these AGNA-positive sera (Graus et al., 2005; Sabater et al., 2008). SOX antibodies have become useful markers of SCLC in these PNDs, most notably in LEMS, where SOX1 and SOX2 antibodies have been found in 64–78% of patients with SCLC and LEMS compared with only 0–5% in non-tumour LEMS patients (Titulaer et al., 2009; Sabater et al., 2008). However, analysis of smaller numbers of patients with other PNS associated with SCLC, such as SSN, PCD and LE (Titulaer et al., 2009; Sabater et al., 2013; Tschernatsch et al., 2010) has indicated that SOX immunoreactivity in these paraneoplastic neurological presentations may be no more common than that seen in patients with SCLC without an associated PNS.

SOX1 and SOX2 antigens share significant sequence homology principally through the high mobility group (HMG) box, a 79 amino acid DNA-binding domain. Identical immunoreactivity to SOX1 and SOX2 in phage clones was detected in 7 of 17 SCLC patients in one study, where SEREX screening of clones from SCLC cell lines showed that the most frequently isolated gene was SOX2 (Güre et al., 2000). In a similar phage plaque assay, Vural and colleagues detected antibodies to SOX1 in 28% and SOX2 in 22% of 90 SCLC patients without neurological symptoms (Vural et al., 2005). In a large series of patients with LEMS, SOX1 antibodies were found in 28/43 (65%) and SOX2 antibodies in 29/43 (67%) of patients with associated SCLC (Titulaer et al., 2009). In this study, we found only two patients (one LEMS-SCLC patient and one non-tumour LEMS patient) had positive SOX2 antibodies but negative SOX1 antibodies, and all positive LEMS samples were confirmed by Western blotting to purified recombinant SOX antigen as previously described (Titulaer et al., 2009). These results highlight the similar immunoreactivity of SOX1 and SOX2 proteins, and their equal value in predicting an underlying SCLC in patients with LEMS.

The level of SOX2 positivity in patients with LEMS-SCLC (37/61, 61%) was higher than that seen in all other paraneoplastic disorders associated with SCLC (OMS-SCLC 3/6, 50%; LE-SCLC 5/12, 42%; SSN-SCLC 16/32, 50%): although similar levels of SOX2 positivity was found in PCD-SCLC (10/17, 59%), where all of these patients also had LEMS. SOX2 positivity was higher in all patients with PNDs associated with SCLC compared to SCLC patients without neurological symptoms.

The reason why SOX2 antibodies are found more commonly in patients with LEMS-SCLC, compared with other PNDs associated with SCLC is not clear. In a previous study published by some of the authors (Titulaer et al., 2009), there was a similar level of SOX2 positivity of about 70% found in both LEMS-SCLC and a small group of patients with SCLC, ataxia and VGCC antibodies, without LEMS, indicating that perhaps it was the presence of pathogenic VGCC antibodies that was co-segregating with SOX antibodies. Moreover, among our 259 control patients with SCLC without neurological symptoms, there were 11 patients who had raised VGCC antibodies, in the absence of LEMS: positivity for SOX2 antibodies in these patients was low (3/11, 27%), similar to the levels of SOX2 positivity seen in the control SCLC population as a whole. Whereas the VGCCs to which specific VGCC antibodies seen in LEMS bind are membrane-associated, the SOXB1 proteins are intracellular, and the likelihood of cross-reactivity occurring through epitope spreading would be low, unless perhaps exposure to the SOX intracellular proteins occurred within the SCLC through cancer cell breakdown and necrosis. The high rate of SOX2 positivity in LEMS and LEMS-PCD may indicate that within SCLC cells, the SOX2 proteins may couple with the intracellular domains of the VGCC complex: although the cellular localisation of SOX2 is thought to be intranuclear, studies in adult stem cells have demonstrated SOX2 proteins principally in the cytoplasm, including possible cell surface distribution (Zuk, 2009). What is known from transcriptional studies is that SOX2 readily forms protein complexes with other proteins (Kiefer, 2007), and it may be that this occurs more frequently with VGCC subunits, rather than Hu-antigen. To date, we have yet to demonstrate similar immunofluorescence staining patterns on cerebellar sections from LEMS patients' sera, or commercial antibodies to VGCC subunits, compared to that seen in the Bergmann glia, typical of SOX antibody staining (Paul Gozzard, Bethan Lang, unpublished data) (Graus et al., 2005).

Eight samples (1.9%) from healthy controls were positive (above mean values plus 3 SD) for SOX2 antibodies at relatively low titres: limited clinical follow-up on local electronic health records to date has not revealed any incident tumours in these subjects, although it is noteworthy that most (5/8) were smokers. Additional sera for western blotting were not available.

SOX2 is highly expressed in SCLCs and is markedly immunogenic in these tumours (Güre et al., 2000). Although SOX2 is also expressed in immature teratomas (Phi et al., 2007; Nonaka, 2009; Liang et al., 2015), thymomas (but not normal thymic tissue) (Cimpean et al., 2011), ovarian adenocarcinomas (Ye et al., 2011) and also breast cancer, particularly in early stage breast lesions (Lengerke et al., 2011; Leis et al., 2012), we failed to demonstrate a significant immune response to SOX2 antigens in patients with tumours other than SCLC, whether these tumours were seen in the context of a PND or not. This may be because SCLC is more highly immunogenic than these other tumours and more capable of eliciting an adaptive immune response to tumour-associated antigens.

We have demonstrated that SOX2 antibodies are highly specific for the presence of SCLC in patients presenting with a range of PNDs. SOX2 antibodies are not usually found in association with other tumours such as teratoma, breast or ovarian cancer, whether a neurological paraneoplastic disorder is present or not. Likewise, SOX2 antibodies are not encountered in patients with clinical presentations similar to classical PNDs such as subacute ataxia or limbic encephalitis, without associated tumours. Therefore, a positive test for SOX2 antibodies in patients presenting with OMS, subacute ataxia, LE or LEMS is highly suggestive of an underlying SCLC. Antibodies to other tumour-associated antigens associated with other malignancies such as breast or ovarian cancer (Storr et al., 2006; Martin et al., 2011) may be useful biomarkers at first presentation in similar PNDs.

## Conflicts of interest

Dr. Lang is a co-applicant and receives royalties on patent application WO/2010/046716 entitled ‘Neurological Autoimmune Disorders’. The patent has been licensed to Euroimmun AG for the development of assays for LGI1 and other VGKC-complex antibodies.
Dr. Titulaer received research funds for serving on a scientific advisory board of MedImmune LLC., for consultation at Guidepoint Global LLC, and an unrestricted research grant from Euroimmun AG. Erasmus University Medical Center has filed a patent for methods for typing neurological disorders and cancer, and devices for use therein. Dr. Graus received a licensing fee from Euroimmun for the use of IgLON5 as an autoantibody test.

## Funding

PG was supported by a fellowship grant from Myaware (UK). MT was supported by a KWF fellowship 2009-4451 by the Dutch Cancer Society.

## Appendix A Supplementary data

Supplementary table Image 1
