# Supplementary material for: The utility of anti-SOX2 antibodies for cancer prediction in patients with paraneoplastic neurological disorders
Source: J Neuroimmunol. 2019 Jan 15;326:14–8. doi: 10.1016/j.jneuroim.2018.11.003 (PMC6375907; doi:10.1016/j.jneuroim.2018.11.003)
Supplement: Supplementary file 1 — Supplementary table [file mmc1.docx]

**The utility of anti-SOX2 antibodies for cancer prediction in patients with paraneoplastic neurological disorders.**

**Supplementary data**

**Supplementary table 1.** Features of patients with small-cell lung cancer.

|  |  |  | SOX2 |  | SOX2 |  |  |
| --- | --- | --- | --- | --- | --- | --- | --- |
|  |  |  | positive |  | Negative |  | P value |
| LEMS-SCLC | |  |  |  |  |  |  |
|  | Female |  | 14/37 |  | 7/24 |  | 0.58 |
|  | median age |  | 60 |  | 61 |  | 0.58 |
|  | %limited SCLC |  | 21/34 |  | 13/21 |  | 0.77 |
|  |  |  |  |  |  |  |  |
| OMS-SCLC | |  |  |  |  |  |  |
|  | Female |  | 1/3 |  | 0/3 |  | 0.99 |
|  | median age |  | 58 |  | 54 |  | 0.96 |
|  | %limited SCLC |  | U/A |  | U/A |  | U/A |
|  |  |  |  |  |  |  |  |
| PCD-SCLC | |  |  |  |  |  |  |
|  | Female |  | 4/10 |  | 4/7 |  | 0.63 |
|  | median age |  | 60 |  | 65 |  | 0.62 |
|  | %limited SCLC |  | 7/8 |  | 3/7 |  | 0.11 |
|  |  |  |  |  |  |  |  |
| SSN-SCLC | |  |  |  |  |  |  |
|  | Female |  | 10/16 |  | 11/16 |  | 0.99 |
|  | median age |  | 66 |  | 67.5 |  | 0.28 |
|  | %limited SCLC |  | 3/8 |  | 6/10 |  | 0.63 |
|  |  |  |  |  |  |  |  |
| LE-SCLC |  |  |  |  |  |  |  |
|  | Female |  | 1/5 |  | 3/7 |  | 0.57 |
|  | median age |  | 63 |  | 65 |  | 0.91 |
|  | %limited SCLC |  | U/A |  | U/A |  | U/A |
|  |  |  |  |  |  |  |  |
| SCLC-no PND | |  |  |  |  |  |  |
|  | Female |  | 37/77 |  | 90/180 |  | 0.78 |
|  | median age |  | 63 |  | 67 |  | 0.02 |
|  | %limited SCLC |  | 25/77 |  | 70/180 |  | 0.39 |

LE = limbic encephalitis; LEMS = Lambert-Eaton myasthenic syndrome; OMS = opsoclonus-myoclonus syndrome; PCD = paraneoplastic cerebellar degeneration; PND = paraneoplastic neurological disorder; SCLC = small-cell lung cancer; SSN = subacute sensory neuronopathy; U/A = data unavailable.
